# Coping with Emotionally Challenging Research: Developing a Strategic Approach to Researcher Wellbeing

**DOI:** 10.1007/s10805-025-09665-5

**Published:** 2025-08-25

**Authors:** Tina Skinner, Kristine Brance, Sarah Halligan, Emily Tsang, Heather Girling

**Affiliations:** 1 Department of Social and Policy Sciences, University of Bath, Bath, UK; 2 Faculty of Education and Health Sciences, University of Limerick, Limerick, Ireland; 3 Department of Psychology, University of Bath, Bath, UK; 4 Safety, Health & Employee Wellbeing Department, University of Bath, Bath, UK

**Keywords:** Emotional, Research, Distress, Vicarious trauma, Secondary trauma, Coping, Wellbeing

## Abstract

While previous work has highlighted the possible impacts of undertaking emotionally challenging research, it is only recently, particularly within the UK with the 2028 Research Excellence Framework focus on research culture, that this subject is starting to gain senior leadership attention. Funded by the UK Research and Innovation, Researcher England, Enhancing Research Culture fund. We undertook an in-depth study involving researchers across topics and disciplines in the humanities and social science, with the objectives of establishing: the impacts of studying emotionally challenging topics on researchers, what they currently found helpful in preventing and/or coping with these impacts, and what additional support they wanted. In this paper we report on findings related to the latter two objectives to provide insight into how future research projects could be ethically designed to minimize distress, secondary and vicarious trauma in researchers. We then use these findings to formulate an innovative strategic institutional response to researcher wellbeing and emotionally challenging studies that can be implemented in three stages: *Bronze*, which is focused on awareness raising and the development of policies and guidance that are built into ethical procedures; *Silver*, involving the establishment of training, clear referral pathways, and (funded) Researcher Wellbeing Plans - including regular academic supervision, team working, and extra time in workloads to undertake wellbeing interventions - built into the design of projects; and *Gold*, a wholistic institutional response where, in addition to the above, policies, processes, practices and culture are proactively attentive to the prevention of and provision for distress relating to emotionally challenging research.

## Introduction

In 2020 the *Journal of Academic Ethics* published an article by Williamson et al. that made more public a conversation many researchers had been having behind closed doors and only with trusted family, friends and colleagues. Theirs was part of a trickle (now thankfully a stream) of articles acknowledging the emotional burden that researchers face due to their topics (e.g., on gender-based violence, see Campbell, 2002; on health research, see Dickson-Swift et al., 2006; on mental health, see Bennett et al., 2025; on the Holocaust, see Goldenberg, 2002). Whilst most of these are, like Williamson et al., from individuals and teams who are grappling with emotionally challenging research, other small-scale studies show us the results of interviews or surveys and/or workshops both within a topic (e.g., bereavement, Batey & Szedlak, 2024) or across topics (e.g., Zschomler et al., 2023). Our own cross-disciplinary/topic research has established that, although most staff undertaking emotionally challenging studies are positive and passionate about their research, they also experience difficult symptoms, such as trouble concentrating, negative emotions, distressing thoughts, gaps in memory, feeling jumpy, reliving the experiences of others, and/or avoiding people/places linked to their research (Skinner, Brance, et al., 2023).

In our research we also asked what participants had in place already in terms of support, and what they ideally wanted in the future. Here, we outline our findings, including that a very small minority of individuals had rudimentary wellbeing plans in place, most of which were reactive, in response to deteriorating mental health, and were researcher-instigated rather than organisational/management/supervisor-led. What participants wanted was a toolbox of options that can be adapted to the individual. Our major contribution is to take these findings and formulate them into a strategic response that has the potential to change how research projects are designed and to transform the institutional culture within which research is undertaken. We start, however, with an overview of existing literature.

### Literature Review

Existing publications have provided multiple recommendations as to how research-related distress might be addressed. Here we review the potential mechanisms and interventions suggested to help researchers cope at three levels: organisation wide; academic supervisors and teams; and individual (i.e., what the researcher can do themselves).

#### Organisation Wide Mechanisms and Interventions

The need for organisations to take responsibility for their staff/students and facilitate support is an important theme in the literature. van der Merwe and Hunt (2019, p.16) highlight the imperative for an “organisational ethos” that fully supports its researchers, including addressing their emotional requirements and the psychological consequences of their work. A supportive and inclusive work culture is generally agreed to be an effective way to improve researcher wellbeing (Bonach & Heckert, 2012; Dickson-Swift et al., 2007; Garrels et al., 2022; Perron & Hiltz, 2006; SVRI, 2015; Zschomler et al., 2023). Yet a common theme across the literature is a lack of emotional support from organisations (Kennedy et al., 2014; Perron & Hiltz, 2006).

Structural changes have been recommended, including: advocating for secondary and vicarious trauma-informed champions to spearhead change (Skinner, Brance, et al., 2023; Zschomler et al., 2023); embedding of clear guidance and protocols aimed at maintaining the wellbeing of researchers doing emotionally challenging work (SVRI, 2015); modifying guidelines/forms of ethics committees to include explicit information on mental health impacts on researchers and responses to those (Dickson-Swift et al., 2007; Garrels et al., 2022; Kiyimba & O’Reilly, 2016; Parker & O’Reilly, 2013); and wellbeing plans for researcher before they start their research (Skinner, Brance, et al., 2023; Zschomler et al., 2023; Garrels et al., 2022). Specific requirements in relation to risk assessment and safety planning are also recommended (Coles et al., 2014; Dickson-Swift et al., 2007; Parker & O’Reilly, 2013; SVRI, 2015; Whitt-Woosley & Sprang, 2018), indeed this is a legal requirement within the UK (under the Health and Safety at Work etc. Act 1974 and Management of Health and Safety at Work Regulations 1999) and European Union. It is suggested this could include: screening for researchers at high-risk (e.g., due to their own personal histories, SVRI, 2015), determining the potential level or risk presented by the topic/activities, and developing procedures to prevent and alleviate distress (Dickson-Swift et al., 2007; Brance and Skinner, 2024a) such as reducing exposure to potentially traumatic data by rotating tasks across a team and limiting numbers of interviews each day (SVRI, 2015).

The need for organisations to provide training related to researcher wellbeing (Bonach & Heckert, 2012; Dickson-Swift et al., 2008; Garrels et al., 2022; Kennedy et al., 2014; Parker & O’Reilly, 2013; SVRI, 2015; van der Merwe & Hunt, 2019; Whitt-Woosley & Sprang, 2018) for academic supervisors/managers and researchers (Parker & O’Reilly, 2013; Velardo & Elliott, 2021) is also highlighted in the literature. Training recommendations include on: safety protocols; building knowledge about the potential emotional consequences of research; recognizing warning signs of impacts in self and others; healthy coping mechanisms; services to access; the particular research methods to be used; and applying emotional sensitivity and appropriate boundaries when interacting with participants (see for examples Bonach & Heckert, 2012; Coles et al., 2014; Dickson-Swift et al., 2006; Garrels et al., 2022; Kennedy et al., 2014; Kiyimba & O’Reilly, 2016; Skinner, Brance, et al., 2023; SVRI, 2015; Silverio et al., 2022; Whitt-Woosley & Sprang, 2018).

Whereas trauma-focused psychotherapy is suggested for researchers who are already struggling, existing literature has proposed that supervision by a ‘clinical psychologist’ (van der Merwe & Hunt, 2019), or ‘clinical supervision’ (Skinner, Brance, et al., 2023; Williamson et al., 2020), ‘professional supervision’ or ‘independent supervision’ (Dickson-Swift et al., 2008), could be used proactively to prevent or mitigate research-related distress and the development of symptoms of vicarious and/or secondary trauma. For clarity, we suggest that *independent wellbeing supervision* would be a clearer term to use to avoid confusion. What we mean by this is a suitable (in terms of training/experience) counsellor or psychotherapist or psychologist, who is paid to provide regular, 1:1, confidential meetings with the researcher that are focused on researcher wellbeing and are independent of (but funded by) the employer and/or research project.^1^ However, the available evidence suggests that such support is rarely accessible (Bonach & Heckert, 2012; Skinner, Brance, et al., 2023; Williamson et al., 2020). Williamson et al. (2020) argue that additional funding for those who require such support would only be a small percentage of the total project grant, and note the potentially significant costs of losing or damaging highly experienced staff. Kennedy et al. (2014) also propose that establishing a support network of experienced and novice researchers can be helpful for alleviating emotional impacts, as advice on how to process and manage the negative emotions can be shared (see also Coles et al., 2014; Skinner, Brance, et al., 2023; Zschomler et al., 2023).

As indicated above, several authors have argued that additional funding should be made available by organisations to support researcher wellbeing (e.g., Dickson-Swift et al., 2008). Beyond this, Skinner, Bloomfield-Utting, et al. (2023) call for both employing and funding organisations to have “a well-funded, detailed institutional strategy focused on prevention, identification, and provision” to address secondary and vicarious trauma and enable the various interventions (mentioned above and below) to be planned and budgeted for accordingly within research projects (see also Skinner, Brance, et al., 2023; SVRI, 2015). Zschomler et al. (2023) indicate that funders can lead by example, providing not only ringfenced funding, but also the good practice guidance and support that is required to facilitate the cultural shift needed in the sector. In this way, the funding and employing organisations could help instigate change, and in the UK this has started in some universities (e.g., University of Bath) through the focus of the Research Excellence Framework 2028 on research culture (see UKRI, 2023).

#### Academic Supervisors and Teams

Regular one-to-one academic supervision meetings are recommended to consolidate supervisory relationships, monitor workloads and reduce emotional distress (Kennedy et al., 2014; Skinner, Brance, et al., 2023; Zschomler et al., 2023), with Silverio et al. (2022) suggesting these occur weekly or fortnightly. Supervisors are advised to play a nurturing role, and to have sufficient knowledge of mechanisms and interventions to identify and address potential research-related distress proactively (Bonach & Heckert, 2012; SVRI, 2015; Velardo & Elliott, 2021; Zschomler et al., 2023), as well as helping researchers develop their wellbeing plans (Skinner, Bloomfield-Utting, et al., 2023; Skinner, Brance, et al., 2023). Debrief sessions with academic supervisors that directly address the emotional distress researchers experience at a particular time (e.g., after a difficult interview) have also been recommended (Batey & Szedlak, 2024; Coles et al., 2014; Dickson-Swift et al., 2007; Kiyimba & O’Reilly, 2016; Parker & O’Reilly, 2013; Silverio et al., 2022; Starcher & Stolzenberg, 2020; van der Merwe & Hunt, 2019; Whitt-Woosley & Sprang, 2018). Silverio et al. (2022) suggested that a ‘buddy’ system could be used within a team to monitor the need for such debriefing, this could also reduce the strain on individual supervisors. Setting regular meetings within a research team, including transcribers (Kiyimba & O’Reilly, 2016), is thought to have a positive impact, providing opportunities for staff to share experiences, gain a sense of solidarity, promote understanding, reduce stress and maintain hope that their work can make a difference (Coles et al., 2014; Dickson-Swift et al., 2008; Kennedy et al., 2014; Parker & O’Reilly, 2013; SVRI, 2015; Silverio et al., 2022). Silverio et al. (2022) suggest team academic supervision could occur every four-to-eight-weeks, whereas Ellsberg and Heise (2005) propose weekly team meetings. Additional group interactions are recommended as well, that a supervisor (or organisation) may commission, potentially including informal (e.g., peer led) wellbeing groups (Skinner, Brance, et al., 2023; Zschomler et al., 2023) alongside relaxing social events (SVRI, 2015; Velardo & Elliott, 2021). However, it is acknowledged that academic supervisors and teams alone might not be adequate for severe distress, that the unguided sharing of experiences with co-workers could propagate distress, and that professional independent wellbeing supervisors should be invited to lead debriefing sessions if the level of secondary and/or vicarious trauma is high (van der Merwe & Hunt, 2019). Indeed, to foster prevention of serious mental health problems, it is recommended that professionals are commissioned to run wellbeing groups and one to one independent wellbeing supervision (Skinner, Brance, et al., 2023; Williamson et al., 2020).

#### Individuals

It is acknowledged that researchers have different thresholds (at which distress may begin) and levels of empathy with participants (SVRI, 2015), and may employ varying tactics such as avoidance, emotional detachment, compartmentalisation and rationalisation (Batey & Szedlak, 2024; Kiyimba & O’Reilly, 2016; Parker & O’Reilly, 2013; Williamson et al., 2020) to help protect themselves from the distress induced by emotionally challenging research. Other ‘coping’ mechanisms include alcohol use, or suppressing upsetting thoughts and feelings, each of which could potentially exacerbate distress (SVRI, 2015; Williamson et al., 2020; van der Merwe & Hunt, 2019). However, the literature includes multiple recommendations for positive self-care, including ensuring awareness of ‘risk factors’, signs of not coping, and how to maintain wellbeing (Coles et al., 2014; Rager, 2005; Skinner, Brance, et al., 2023). Practical recommendations for the latter at the individual level include recreational activities, such as exercise, reading, listening to music and interacting with nature and animals (Coles et al., 2014; Goldenberg, 2002; Kennedy et al., 2014; Williamson et al., 2020). Taking breaks, breathing exercises, yoga and mindfulness are also recommended as useful activities to relax the mind and set thoughts about work aside (Coles et al., 2014; Kennedy et al., 2014; SVRI, 2015; Whitt-Woosley & Sprang, 2018). Self-reflexive strategies, such as journalling, have been said to be beneficial, allowing researchers to process and reflect upon their emotions (Fahie, 2014; Goldenberg, 2002; Karcher et al., 2024; Velardo & Elliott, 2021). Having a time slot for self-care every day or implementing scheduled rest breaks have also been proposed (Bonach & Heckert, 2012; Dickson-Swift et al., 2007). Kennedy et al. (2014) suggest that researchers should occasionally remove themselves from stressful environments to protect themselves from the emotional adversity of their work; whereas Silverio et al. (2022) argue that researchers should be removed by managers if they cannot cope. It is also recommended that researchers set boundaries between work and personal life and maintain their work-life balance (Bonach & Heckert, 2012). If individuals do need help, they are encouraged to reach out to supervisors/managers, their organisation and support services (Zschomler et al., 2023).

#### Summary

The growing literature on researcher wellbeing and emotionally challenging topics, as indicated above, provides a wide range of possible coping mechanisms, from the organisational to the individual. However, most studies have not systematically explored these options with researchers. Given wide-ranging and diverse recommendations for support, coupled with tight financial contexts for many organisations, it is essential to identify priorities through consultation with researchers themselves. We addressed this issue by asking researchers directly what they want, informed by the options provided above; and use the resultant information to develop a staged approach for organisations to follow in addressing the wellbeing of researchers working on emotionally challenging topics.

The research questions we address in this paper are:

- What coping mechanisms do researchers undertaking emotionally challenging research already have?
- What additional support do they want?

## Methods

After a favourable ethical opinion was gained from the University of Bath Ethics Committee (Ref: S23 012); the study involved 31 in-depth semi-structured interviews, as well as pre-and-post-interview questionnaires on the survey platform QuestionPro. A purposeful and multi-stage sampling method was applied to select participants. Those eligible for interview were: (i) staff members on a research contract or research and teaching contract, (ii) within the Faculty of Humanities and Social Sciences at the University of Bath, and (iii) engaged in or who had previously undertaken research on potentially emotionally challenging topics. Students were excluded from the study, except lecturers/researchers also undertaking a PhD. Researchers were identified and contacted based on information from the University website from April 19th to April 27th, 2023. We recruited 15 participants following direct invitation based on their research profile (38 invited); 14 based on snowball sampling (29 invited); and two based on a faculty wide invitation.

Before the interview, participants completed a questionnaire capturing demographic information (see Table 1). Nineteen females and 11 males (one participant did not give this information) aged between 25 and 60 years were interviewed, from five different departments, most commonly working as lecturers or senior lecturers. Whilst the sample was diverse in terms of nationality/country of origin, the majority were white; five considered themselves disabled; one indicated they were from the LGBTQi communities.

**Table 1:** Demographic characteristics of the sample

Variable	*N* = 30*
Age	25–60 (M = 42)
Gender	
Female	19
Male	11
Non-binary	0
Disabled	
No	25
Yes	5
Job title	
Professor	3
Reader	3
Senior lecturer	7
Lecturer	8
Research fellow	1
Research associate	5
Research assistant	2

* One participant did not complete the pre-interview questionnaire

The interviews were shaped by a flexible topic guide, with 30 conducted via Microsoft Teams and one face-to-face. Interviews, on average, lasted 54 min and were professionally transcribed. Qualitative data were analysed thematically, following the six steps outlined by Braun and Clarke (2006), with analyses recorded in NVivo 14. These stages were: (1) familiarising with the data through recordings, transcriptions and notes; (2) generating initial codes systematically across the dataset while closely examining transcripts; (3) collating the codes into potential themes in a codebook; (4) reviewing and refining these themes collaboratively, and creating a thematic map; (5) further defining and naming themes; (6) selecting key extracts to convey the core arguments and themes effectively. In addition, to ensure coding trustworthiness (Korstjens & Moser, 2018; Lincion & Guba, 1985), investigator triangulation was used at each of the above stages. First, all the interviews were listened to by co-authors Brance and Skinner. They then independently coded five interviews to develop the codebook. Coder agreement was high, and any differences were resolved by discussion before the codes were applied to all transcripts by Brance. Any new codes developed in subsequent coding, or uncertainty about coding of a section of transcript, was also discussed by Brance and Skinner and added to the codebook. Analysis and selection of quotes was checked by co-author Halligan. Credibility of the research was further enhanced through prolonged engagement, in that the authors work in the same organisation as the participants and are highly familiar with the context under investigation. Member checks were made through several avenues, including participants being able to feedback on the project findings before general release.

Of course there are limitations of the data. First, interviewees mentioned different ways that they tried to cope with their research related distress, as well as things that they did not currently have but thought might help them, but they did not always indicate which they would prioritise if they were given the choice. Second, many of the researchers had limited ideas in relation to what might help them as they had never discussed the issue before. This initially limited our ability to draw conclusions about preferences relating to coping and support. Therefore, to maximise our capacity to make recommendations to improve coping practices and support services, we designed a follow-up questionnaire with 59 different support/coping options that had been given in both the interviews and existing literature outlined above, and asked participants to rate their preferences on a 5-point Likert scale ranging from 1 (not at all helpful) to 5 (extremely helpful). From these data we generated descriptive statistics to provide further trustworthiness checks for the qualitative analysis through data triangulation (Korstjens & Moser, 2018). Due to the practical reasons of space and the potential for institutions to be overwhelmed by to many suggestions, we have included support/coping mechanisms in the findings section and the recommendations if: it was indicated in the interview by more than one person as important; it was ranked as helpful or extremely helpful by 10 or more people in the quantitative data; and there was supporting evidence in the existing literature that it could be helpful for emotionally challenging research. This means that other possible coping mechanisms/supports are not included (for example independent legal advice only made one of these criteria so is not included in the recommendations though it could be very helpful and is now provided by the University of Bath). This links to a third limitation: the literature we reviewed, the suggestions from participants, and our research team’s experience each relate to existing researcher’s knowledge on how to address researcher wellbeing and emotionally challenging topics. We are not, for example, psychotherapists, psychiatrists or medical doctors. Thus, there could be coping mechanisms and interventions that are more or equally effective compared to those we discuss here. Fourthly, the study was focused within the Humanities and Social Sciences Faculty at the University of Bath in England. This University is, for example, ranked 132nd in the QS World University Rankings (QS TopUniversities, 2025), it is research focused, relatively well-resourced, and generally has a lower teaching load than institutions that became universities in the UK post-1992. Different universities, disciplines and nations may have varying cultures, approaches, pressures, experiences and specific needs. Fifth, the authors are also University of Bath staff. This could have led to participants being reluctant to share information with us. We addressed this from the start by offering participants the option to choose an interviewer they felt most comfortable with and providing repeated reassurance about anonymity. Indeed, we found it to be an advantage to be an ‘insider’ in terms of gaining access, encouraging participants to open up, and providing an existing understanding of the institution. Finally, it is also important to acknowledge that, since the research was undertaken in 2023, the University of Bath has worked to improve their research culture including the experiences of staff undertaking emotionally challenging research (e.g., training on this issue is now an option in mainstream training for staff and PhD students). Thus, the data does not necessarily reflect present day staff views or the current practices at the University.

## Qualitative Findings

The qualitative data indicates that participants employed various coping mechanisms, mainly informal, with only a minority accessing formal support funded by their organisation. The overwhelming perception was that there needed to be more support for staff, but that there should not be a “one-size-fits-all” (Participant 13) approach. In the following sub-sections, we discuss these mechanisms under the headings: *organisational culture*,* support and guidance*; *academic supervision/management*; *colleagues*,* teams*,* groups and networks*; *independent wellbeing supervision*,* counselling/therapy and mentors*; and *individual coping mechanisms* (e.g., exercise, hobbies).

### Organisational Culture, Support and Guidance

There was variation in participant attitudes towards the existing organisational culture, linked to factors including academic career stage, level of control they had over their work (e.g., contract researcher vs. principle investigator), years at the University, the supportiveness of the team they worked in, or other variables (e.g., other pressures, such as being a carer or being disabled or otherwise marginalised). Some participants felt that they were already in a supportive research culture where they could have “vulnerable conversations” (Participant 29), expressed gratitude for the support they received, or highlighted that the academic environment has seen positive shifts in attitudes towards wellbeing. Participant 25 reflected on this perceived change, stating: “It is much more accepted, I think, for people to say, I’m really struggling right now, or this has really bothered me. I grew up as an academic in a culture where that wasn’t valued.” However, such positive perceptions about the culture were not widespread. Respondents stated that they rarely discussed their wellbeing needs in relation to their research at work. A significant reason for this was the perceived absence of formal support mechanisms. Some believed that the University did not offer such mechanisms. Others acknowledged the existence of support at the University but did not seek it out, with Participant 13 stating: “I might have had to more proactively go out and ask for it”.

A reluctance to access University services was, for some, tied to a deeper mistrust of the organisation: “I don’t think we really trusted the organisational culture to help us” (Participant 16), “to be honest, I don’t trust the University in terms of […] seriously taking care of mental health” (Participant 21). Staff suggested that the University should work towards a research culture that recognises, acknowledges, and openly addresses the possible impacts of engaging in potentially distressing work.

“I don’t know that the University is as responsible as it could be, in terms of protecting its researchers overall, so not just in terms of the emotional consequences of doing particular forms of research, but also, you know, in terms of providing the resources and the facilities to do good research, to you know… even producing the research culture that allows people to debrief and discuss research.“ (Participant 31).

Another participant stated:

“In an ideal world, […] the University would feel like a sort of kind parent […] that you could turn to, when you are distressed, who you feel looks after you. But I don’t really actually think the University feels like that right now. It doesn’t feel like a kind parent. It feels like quite a parent that you wouldn’t want to turn to where if you had problems, and especially emotional ones”. (Participant 16)

Participants thought that cultural change was needed to move away from what was seen by most as a prevailing perception that admitting to the toll research takes on one’s mental health may be perceived as a sign of weakness, raising doubts about one’s suitability for a career in a particular research area: “Because it’s not seen as normal, you know you’re kind of almost admitting that you’re struggling and that you’re a failure, and you’ve got some weakness” (Participant 22).

Another reason given for not seeking support was the belief that it was the University’s responsibility to offer these services and inform the staff proactively. Indeed, interviews indicated that any formal support that was occasionally available was reactive rather than proactive. What participants wanted was proactive support. As Participant 13 states, such support for researchers should not be *“one size fits all”* but rather:

a toolbox of options that are available to researchers in a flexible way according to their needs […] both informal and formal mechanisms within each research team or the wider department or an organisation. But also that there are those independent people, expert people to go and talk to in more depth if required.

Staff were frustrated by the lack of substantive University guidance, support, training and funding in this regard. They were particularly annoyed by what they saw as tokenistic individualizing interventions: “I get really frustrated about e-mails telling me about the latest XXX […] which puts all the blame on me” (Participant 21). Despite recognising increased discussions about staff wellbeing at the University, it was often viewed as a one-off (e.g., standalone wellbeing sessions) that was without genuine commitment or adequate funding. Participants wanted the University to put “their money where their mouth is” (Participant 2).

Further organisation-wide issues identified in the data included the need for effective risk assessments, and clear written guidance, processes and pathways of referral. Risk assessment and safety planning, despite being a legal requirement, were viewed as a “ticking boxes exercise, […to] make sure that the University has the right paperwork rather than a genuine concern in how I cope” (Participant 21). There was an almost complete lack of awareness of the existing prompts to consider mental health in the University’s risk assessment form. Without adequate awareness and risk assessment, mental health needs were often not discussed:

“at no stage did anyone say to us, ‘you guys might want to keep in mind that this could be something you yourselves might need support for’. There was a lot of focus on what will you do if your participants become distressed; lots of focus on that. But nobody said to us, what if you guys become distressed?” (Participant 16).

Thus, things like the need to have a private office so that distressing work was not “polluting” their homes (Participant 22), or extra time in workloads for managing emotionally challenging research were rarely planned for. Despite this, the advantages of such planning were highlighted (primarily in hindsight) by participants, for example: “it’s quite useful to have a break between doing interviews, so that you have time to reflect on that and think about that naturally, do something about it if you need to, for your own health and wellbeing” (Participant 30).

One of the dangers in not doing a risk assessment - beyond “ticking boxes” - and providing appropriate support was illustrated by Participant 22 when she stated “there is some collateral damage on my mental and emotional wellbeing”. She goes on to say:

I’ve been doing a whole load of questioning, I would say, since this XXX project. […] So I’ve been, for the last two years very proactive about applying for jobs outside of academia. […] Because I just think if this is the future, it’s not worth it.

### Academic Supervision/Management

Academic supervisors and line managers were perceived to hold an important role in researcher wellbeing. Researchers reported benefits when they were supported by their supervisors/managers. Having the opportunity to talk with supervisors or other team members was indicated as helpful to offload emotions and research challenges. However, participants desired more frequent supervision and the chance for more structured debriefing mechanisms. Notably, these tended to be early career researchers or those involved in particularly sensitive research tasks, such as interviewing and transcribing, for example: “I think it would be nice to have like some sort of debriefing or something like this! You know, so being able to talk about the experience” (Participant 21). Some more senior participants emphasised that part of their role as managers/supervisors was to plan down time for their staff when they were undertaking emotionally difficult tasks (Participant 25) and encourage them to have breaks when necessary and talk to them when needed. For example: “…I was with them […] to ask them how they’re doing and what they do outside of their work and if I see them stressed, then telling them to take some breaks” (Participant 24). At the same time, staff also suggested that supervisors/managers should respect the privacy of their researchers, understand that distress thresholds vary among individuals, accept that some researchers may prefer not to share sensitive matters with them, and understand their own limitations in helping the staff they manage.

### Colleagues, Teams, Groups and Networks

Over half the interview participants emphasized the importance of trusted colleagues as support sources, whether within their research group, the broader University, or networks developed nationally or internationally. A reason identified for why colleagues were thought to be particularly helpful was the perceived understanding of common issues they were facing. Just under a third of interviewees had a group of colleagues working on a particular project, or simply the same topic, that informally came together to form a support group, for instance:

“And the only thing that really happened for us was we kind of formed our own mini-support group. As in, we just talked to each other. We would meet for coffee. And we would just chat about how this was going for us, which we all found hard” (Participant 16).

Not everyone had this, for instance, one participant described a detrimental research team that negatively affected their wellbeing and added extra work. Another participant lacked a supportive environment, with no one knowing their activities or checking in on them during fieldwork. When they attempted to address specific issues, they perceived that their experiences were dismissed by colleagues. Indeed, fostering a supportive team can be challenging for supervisors:

“I know what I’ve tried to foster, within my, my PhD students. I have tried to foster kind of a sense of community where they kind of trust each other, they can talk to each other, they can talk to me that we can all feel kind of comfortable sharing our difficulties, but […] it is difficult” (Participant 2).

However, participants suggested that adopting a team-based approach can mitigate the emotional strain of their research. Participant 11 made sure “I’ve always worked in a team” to avoid the isolation that can come with emotionally challenging work, but also to ensure “not everything hinges on me, so if I […] just can’t do it, they have someone else to just step in”. Individuals who reported a more supportive environment, where team meetings, joint data coding/analysis and (informal) debriefing were common, were generally more positive about their working environment and wellbeing. For example:

“We have monthly meetings currently, and more often if [... we need] them in between, […] we have those fixed in the diary, if we don’t need them we cancel them, […] And particularly when […we start] getting the data, […] then we start looking at the data together, that is really helpful to debrief on” (Participant 30).

Participants also suggest that proactively fostered connections between researchers undertaking distressing research beyond just their research team would be helpful in order to learn “from other people about what they’re doing and how they’ve managed certain things that have gone wrong or might go wrong” (Participant 30), and have “more time and space to have to talk about these things and reflect with people that value the thing that you’re doing and understand those impacts” (Participant15). Participant 27 talked of a need for a *“*safe space”, and likewise Participant 3 wanted a “safe forum” or “network” where people doing “similar stuff” could “talk confidentially” about their emotionally “difficult work”. Participant 8 referred to such a community as a “marketplace where people kind of share […] how they cope, what they found useful”. Overall, the cultivation of connections among researchers was seen as vital, creating an environment where various conversations unfold, often revolving around the effects of research, the inherent challenges, and how to cope with them.

### Independent Wellbeing Supervision, Counselling/Therapy and Mentors

Three participants had pre-arranged independent wellbeing supervision by a psychologist or counsellor to help support their research work. An additional two gained this support because they experienced such significant distress from their research that they requested this service. Such professional psychological support was recognised as necessary by participating research staff and academic supervisors/managers. The reason for this was indicated by Participant 25, who supervises projects:

Whilst we have all the debriefs and everything, I am not clinically trained to either diagnose trauma or to diagnose mental health issues or declining mental health. And whilst I hope they would come to me if they were feeling traumatised or feeling like it was having an effect, I am very conscious of the fact that […] I’m their line manager, and they may not want to share that with me, or they may worry about… I hope they don’t, but they may worry about what conclusions I will draw about their competency or skill.

Indeed, staff indicated independent wellbeing supervision could be helpful in addressing concerns arising from potentially distressing research even if they did not currently have it. For example: “Because it’s kind of like downloading that emotional content somewhere, so it’s not stuck in your head and not going round your mind all the time, which is not very helpful!” (Participant 30).

In addition, several participants mentioned therapy or counselling as a current or possible coping mechanism. Most interviewees indicated reluctance to utilize the standard University counselling services for profound or prolonged issues stemming from their research. Some common reasons for the reluctance included the limited number of sessions available, a lack of trust in these services, an absence of follow-up, and generally inadequate awareness about these services. Participant 2 shared, “I did a few years ago, […] the University programme, where you get six sessions with a therapist, but it was too short, I think, and it’s not what I needed. I needed something a bit more long-term”. Several participants paid for private therapy or counselling themselves.

Mentors and coaches were also talked about in interviews, though less so than counselling and independent wellbeing supervision. While some only really wanted to talk to those with expertise in their research area, others wanted mentoring and/or coaching irrespective of the mentor/coach’s experience:

“Some form of mentoring system would be really helpful […] someone who doesn’t have to be an expert in that area, but can be curious enough about that area so they can respond in a way that can deepen the awareness of what’s happening or deepen the understanding.” (Participant 10).

The University currently has both mentoring and coaching schemes, but it does not have mentors who specialise in emotionally challenging research, and there was limited awareness/use of existing schemes among the participants.

### Individual Coping Mechanisms

Several participants saw their ‘predisposition’ as a key foundation for their ability to cope. However, on closer analysis of the data, it was observed that ‘predisposition’ arose from training, accumulated research experience, personal or spiritual or non-research related professional experience, what some respondents perceived to be ‘innate’ qualities (e.g., ability to compartmentalise), not having a shared experience with the research participants, or a combination of these things. Linked to this, a recurring theme in the interviews was acceptance, which manifested in various ways, helping researchers to cope and/or (more negatively) resigning them to cope without University support. This included acknowledging and normalising their emotional and physical responses, accepting the inherent challenges of their research topic, accepting their limited capacity to assist individuals, accepting (though not necessarily happily) the lack of University support, and accepting the eventual need for emotional decompression. For example:

“I think I’m just comfortable with the fact that it’s going to be difficult. And that’s kind of just part of it, because you can’t expect to go into these topics in these populations and like, and [expect] it’s just going to be easy and straightforward” (Participant 14).

Whilst only a small minority had a ‘self-care’ plan in place, all endorsed ways of coping that they tried to or would like to do. Engaging in different types of exercise, such as walking the dog, running, swimming, yoga, and going to the gym, was highlighted as a key coping mechanism. For example, in their interview Participant 1 stated they used a swimming pool to help them to “wash off” their day of interviewing distressed participants before they went home:

“So, for example, I would use the swimming pool. And to decamp from work to home life. So, I’d physically immerse myself in water, and it was no good surface swimming. No, you have to immerse yourself completely, fully, and to literally wash off the day before entering the home life in the evening.” (Participant 1).

Similarly, non-sporting hobbies (e.g., gardening, reading, connecting with nature, having a pet, enjoying music), were considered effective coping mechanisms for all participants. Like exercise, this was emphasised not just as a coping mechanism but also as a way to enhance productivity through a better work-life balance. This formed part of a more general point that several made about getting their ‘work-life’ balance ‘right’ for them: “I’ve also realised since I work towards a better work-life balance that I’m actually far more productive when I’m better within myself rather than when I’m working too much” (Participant 2).

Spirituality/religion provided meaning and support amidst challenges for some participants. This helped them maintain hope and offered a framework to navigate and respond to the complexities of human suffering or other research-related challenges. For example:

“I went on a long kind of learning and spiritual journey through this learning, […] which massively professionally and politically impact and made me much more able to do what it is that I do, healthily and effectively […]” (Participant 29).

Another coping mechanism used was reducing the time they spent with the topic by moving to part-time working or, in the following excerpt, by not being involved in data collection/generation that involves direct stories from distressed participants: “I can do research in an organisation and talk with professional people, but I couldn’t be out on the ground listening to those stories. I just know that I couldn’t do it” (Participant 8). Whilst limiting work in some way, either through the time exposed to it or the type of work they did, was helpful for some participants, there were also less positive forms of avoidance. Participant 22, provided an instance of this when discussing their reaction after they were allocated the detailed analysis of what they knew would be particularly distressing codes within a data set:

“Yeah, it’s called avoidance [of data analysis] by getting up and just going, “oh fuck this, I’m off to [...do a hobby]!” But then equally, doing that classic thing that you’re not really enjoying your time [...doing the hobby] because you feel guilty that you’re not at your desk coding your data!” (Participant 22).

Other ‘coping’ mechanisms could be potentially harmful. Alcohol emerged as a significant concern for a minority of participants, with one participant acknowledging its use as a way to numb their emotions or deal with stress. Others were unable to set limits, taking on more tasks than they could handle, which led to feeling overwhelmed, as a way of appeasing their sense of guilt at the lack of support available for their research participants.

## Quantitative Findings

**Fig. 1:**
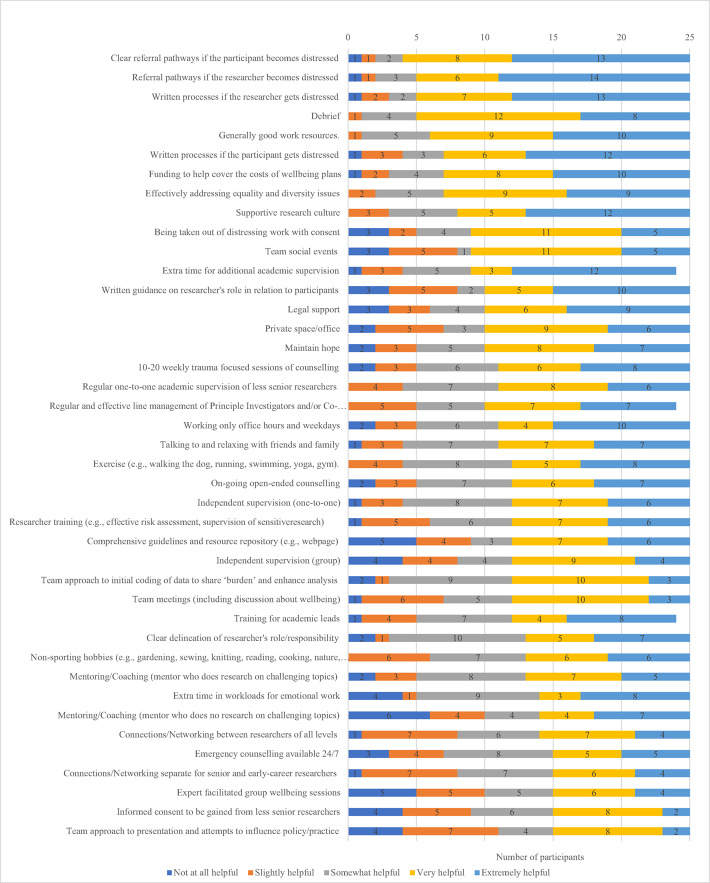
Perceived to be the most helpful support/coping mechanisms/interventions. *Note*: *N* = 25 participants. The figure is ranked first by adding together the numbers of participant finding a mechanisms (potentially) very + extremely helpful and ordering them highest to lowest. If this number is the same for two or more, the mechanism with the highest number finding it (potentially) extremely helpful is placed highest

To help us rank the importance of the 59 coping mechanisms/interventions recommended in the interviews and/or literature we reviewed, we invited participants to complete a questionnaire, 25 respondents did. For reasons of space, we have included in Fig. 1 the coping mechanisms/interventions that were ranked by at least 10 participants as either very or extremely helpful. It is striking that several of the top ranked interventions are creating referral pathways and written guidance for if the researcher or participant gets distressed, alongside developing a supportive research culture. Having good work resources to do their job properly was also highly ranked; this could include not only technology that works, but also private office space so data is not - as one of our interviewees put it - “polluting” (Participant 22) their homes. Funded Researcher Wellbeing Plans were one of the most popular options (above unfunded plans). Such a plan could include a selection (depending on risk assessment and need) of many of the options in Fig. 1. For example, existing University counselling services were seen as (potentially) helpful but the option of 10 to 20 trauma-focused counselling sessions (a service not currently generally available without a specific case being made to HR), independent of the University, was viewed more favorably. Also seen as important was extra-time in workloads to approach this emotional work with the due diligence required; and working only office hours to ensure that they had time to unwind and relax with friends, exercise and do other hobbies.

Regular academic supervision (weekly or fortnightly depending on need) to check in on emotional wellbeing, discuss the previous week’s work, and plan the next week’s work, was viewed as important. The academic supervisor (or a colleague, or an independent wellbeing supervisor) could also provide one of the most popular options which was having the opportunity to debrief (though training on how to debrief would be appropriate if this option were implemented). Other tasks that could be linked to effective academic supervision of emotionally challenging research are indicated in Fig. 1, including: ensuring informed consent was gained from the researcher to undertake their work, temporarily taking researchers out of tasks (with consent) if they are not coping, and maintaining hope that the research can make a difference even if that is just in a small way. Academic supervisors could also lead in the fostering of a sense of community and support through: team meetings/events; a team approach to data coding, analysis, writing up and dissemination; team meetings that include discussing wellbeing; and encouraging staff to access networks beyond their research team if needed. It is also notable that being given extra time to do this level of supervision, and for supervisors to be regularly and effectively line managed themselves, was also seen favorably, as was training to risk assess, safety plan and understand how to look after their own wellbeing and that of others. Having the option to reach out to mentors/coaches was also thought to be helpful.

## Discussion and Recommendations: a Strategic Approach to Researcher Wellbeing

The findings of this research echo the findings and underline many of the recommendations of other small-scale studies that we outlined in the literature review. Of particular importance is changing the research culture to effectively address the possible impacts of emotionally challenging research. This could be best facilitated through organisational, indeed sector-wide, development and funding of a *strategic approach* to *promote* awareness of, and *prevention* of and *provision* of *support* for, distress, secondary and vicarious trauma. Based on our findings, and existing literature, four key areas should be targeted in conjunction: organisational, academic supervision/management, teams and groups, and individuals.

### Organisations

Organisations, including funders and employers, need to enable a supportive research culture that proactively and positively acknowledges and discusses the issue (see also Skinner, Brance, et al., 2023; SVRI, 2015; Zschomler et al., 2023). If a whole organisational approach is not currently feasible this can be achieved in smaller pockets within an organization (e.g., a department or a research team) and/or at least minimum standards can be met. Below we indicate how a supportive research culture can be facilitated:

1. *Awareness raising* (minimum standard) and *leading by example* from the top.
2. *Written processes*,* guidance and pathways* (minimum standard), so staff at all levels know:
  - How to maintain wellbeing and what to do if a researcher needs help (see also Skinner, Bloomfield-Utting, et al., 2023; Skinner & Brance, 2024a; SVRI, 2015; Zschomler et al., 2023).
  - How to complete *risk assessments and safety planning* (see also Brance & Skinner, 2024a; Coles et al., 2014; Dickson-Swift et al., 2007; Parker & O’Reilly, 2013; SVRI, 2015; Whitt-Woosley & Sprang, 2018), with appropriate assistance, advice and monitoring to support these process (e.g., through ethics committees and organisational risk assessment teams).
3. *Specialist training* provided on guidance (see also Skinner, Brance, et al., 2023; SVRI, 2015; Whitt-Woosley & Sprang, 2018), including on the impacts of emotionally challenging topics, how to develop wellbeing plans, effective risk assessment and safety planning, how to protect the wellbeing of participants and researchers, and supervision of emotionally challenging research.
4. *Researcher wellbeing needs embedded in institutional ethical* (see also Dickson-Swift et al., 2007; Garrels et al., 2022; Kiyimba & O’Reilly, 2016; Parker & O’Reilly, 2013) and *funding application processes* (Skinner, Brance, et al., 2023; SVRI, 2015) to prompt researchers routinely to think about the issue and how they will address it. Funders should encourage and be more likely to fund applications that proactively address researcher wellbeing.
5. *A safety net for researchers*: Where projects do not have specific funding for support, employers should seek to provide a safety net of provision (Skinner, Brance, et al., 2023).
6. *Specialist mentoring and/or coaching* for supervising/researching emotionally challenging topics to improve their confidence/knowledge/skills available to run such projects.
7. *Extra time in workload* models to develop and implement wellbeing plans, generate/code/analyse potentially distressing data, for team discussions, dissemination, extra supervision and looking after wellbeing (see also Skinner, Brance, et al., 2023; SVRI, 2015; Zschomler et al., 2023).
8. Ensuring that *resources* are available for researchers to do their jobs effectively and safely (e.g. office space, see also Ellsberg & Heise, 2005; SVRI, 2015).

### Academic Supervisors/Managers

Key to researchers’ experiences was how they were supervised currently or in the past. Given our findings on impacts (Skinner, Brance, et al., 2023) and what currently works or was requested by participants in terms of effective supervision, we recommend academic supervisors/managers:

9. *Risk assess and safety plan (minimum standard)* before applying for funding (Brance & Skinner, 2024a).
10. *Build extra time into projects* to allow for ‘time out’ of stressful tasks and time for wellbeing activities (see also Skinner, Brance, et al., 2023).
11. *Ensure informed consent* of the potential researcher (see also Skinner, Bloonfield-Utting, et al., 2023).
12. *Put in place/source resources*,* activities and services for Researcher Wellbeing Plans* (see also Skinner, Bloomfield-Utting, et al., 2023; SVRI, 2015).
13. *Provide regular and effective academic supervision* (weekly or fortnightly, depending on need, see also Silverio et al., 2022; Skinner, Bloomfield-Utting, et al., 2023; SVRI, 2015) to establish a positive working relationship, establish clear role/responsibilities for staff, discuss the work that week, plan the next week, discuss *Researcher Wellbeing Plans* and share coping strategies; and help maintain hope/expectations that the research can make a difference.
14. *Ensure they have the skills and availability to supervise this work as well as debrief research staff* (see also SVRI, 2015; van der Merwe & Hunt, 2019), potentially linking the latter into a ‘buddy’ system to enable a sharing of the workload (Silverio et al., 2022) or through the independent wellbeing supervisor if the are not able to provide debriefing within the team.
15. *Are themselves sensitively and effectively line managed* by people who are aware of the challenges they face (SVRI, 2015).

### Teams and Groups

Working in effective *teams* and reaching out to talk to colleagues and *groups* of researchers was also something that participants either wanted or currently found helpful (see also SVRI, 2015; Silverio et al., 2022). To facilitate a *team approach* to share the ‘burden’ of this emotional work and build a sense of community (even if the team is just the supervisor and researcher), we recommend supervisors (and researchers) arrange a combination of:

16. Team meetings that include wellbeing (minimum standard).
17. Team data coding/analysing.
18. Team presentations/impact work.
19. Team events (e.g., going to conferences together, group socials).
20. Encouraging researchers to link into existing networks/groups (minimum standard) or develop their own.

### Individual

At the *individual* researcher level we recommend the use of *Researcher Wellbeing Plans* (minimum standard; see Brance & Skinner, 2024b; Skinner & Brance, 2024b; Skinner, Bloomfield-Utting, et al., 2023) adapted to the needs of the project and researcher. What goes into the plan will depend on the risk assessment and the individual researcher’s needs e.g., including a bespoke combination of:

21. Ways to alleviate stress and distress such as taking a break, seeing/thinking about people we love, using the gym, going swimming/running, and other hobbies.
22. Regular academic supervision (minimum standard) weekly or fortnightly when need is greatest.
23. Sources of help such as freely available services (minimum standard).
24. Monthly independent wellbeing supervision by a counsellor or psychotherapist (minimum standard for highly challenging research or particular vulnerable staff, see Brance & Skinner, 2024a).
25. Specialist trauma focused counselling if required.
26. Expert facilitated wellbeing workshops.
27. Team working and network membership.
28. Provision of necessary supports required for wellbeing plans to work, such as extra time in workloads and working only office hours so that sufficient time is available to recharge.

Taken together, such a strategic approach would mean individual researchers are no longer left to fend for themselves, but rather have a Researcher Wellbeing Plan that is imbedded within a positive academic supervisory relationship, effective team management and an organisational culture that proactively encourages, facilitates, funds and supports them to look after their own wellbeing effectively. This is why we call them Researcher Wellbeing Plans, rather than the individualized ‘self-care plans’. Whilst not all staff will use the plans, or services and support, research suggest that even the perception of support being available can ameliorate the negative effects of distress (Thoits, 2011).

It may not be possible for an organisation to implement all of the recommendations at once, so we have formulated a phased approach each building on the other: *Bronze (minimum standards)*, which is focused on awareness raising around the possible impacts of such emotional work and starting to put the above guidance and processes, and basic unfunded Researcher Wellbeing Plans, in place to address it; *Silver*, where practice starts to change through specialist training, effective management/supervision, and the inclusion of funded Researcher Wellbeing Plans in funding applications, so that teams undertaking the most challenging research or researchers with the most need are well catered for; and *Gold* standard *(an achievable aspiration)* where an organisation’s policies, processes, practices and culture are proactively attentive to the prevention of and provision for distress, secondary and vicarious trauma in research, including a safety net for researchers who do not have funded projects but nevertheless require funded Researcher Wellbeing Plans. Some of the most popular options involve written processes and guidance. Thus, some very positive steps can be taken relatively cheaply to achieve Bronze. Funders can lead by example by providing funding for and guidance on what provision is appropriate for enabling researchers to continue undertaking this emotionally challenging work.

## Conclusion

The Researcher Wellbeing Project has identified that academics undertaking potentially distressing research are inspired and driven by the research they do. However, such research can have substantial impacts on the researchers. How well researchers cope with emotionally challenging research is not just about them as individuals. It is about how well they are supported by the culture, mechanisms and interventions they have access to. Thus, individual researchers’ knowledge, skills and experience contribute to their wellbeing, alongside how effective academic supervisors/managers are in designing projects and managing teams/individuals, as well as whether researchers are supported by colleagues and within teams and groups, and how much thought and investment organisations have put into how researchers are looked after. Ideally, organisations that undertake and fund research on emotionally challenging topics need to put plans in place to develop a well-funded strategy focused on *prevention* of and *provision* for distress, vicarious and secondary trauma linked to such research. A phased approach working towards this is an urgent and viable option.

## Resources

### Researcher Wellbeing Project (RWP) Resources

Researcher Wellbeing Project Webpage: This has free resources as well as further details about the project and training we run: https://www.bath.ac.uk/projects/the-researcher-wellbeingproject-rwp-addressing-researcher-distress-trauma-and-secondary-trauma/.

RWP Researcher Wellbeing Plan guidance:

- Researcher Wellbeing Plan guidance: https://www.researchgate.net/publication/380317446_Researcher_Wellbeing_Plan_Guidance.
- Researcher Wellbeing Plan Template: https://www.researchgate.net/publication/380317302_Researcher_Wellbeing_Plan_Template_C.
- Independent wellbeing supervisors (‘clinical supervisor’) who specialise in vicarious and secondary trauma.

RWP guidance for researchers if participants experience distress:

- Guidance for researchers if participants experience mild or moderate distress: https://www.researchgate.net/publication/380317624_Guidance_for_researchers_if_participants_experience_mild_or_moderate_distress.
- Guidance for researchers if participants are at risk of serious harm: https://www.researchgate.net/publication/380317620_Guidance_for_researchers_if_participants_are_at_risk_of_serious_harm.

Other RWP guidance:

- Guidance for what to do if a researcher/you get(s) distressed: https://www.researchgate.net/publication/380374975_Guidance_on_what_to_do_if_a_researcher_gets_distressed_during_emotionally_challenging_r.
- Guidance for institutional support of emotionally challenging research (funders and employers): https://www.researchgate.net/publication/380317583_Guidance_for_institutional_support_of_emotionally_challenging_research_funders_and_empl.
- Risk assessment guidance for emotionally challenging research: https://www.researchgate.net/publication/380317406_Risk_assessment_guidance_for_emotionally_challenging_research.
- Wellbeing guidance for researchers, teams and supervisors: gold, silver and bronze: https://www.researchgate.net/publication/380317102_Wellbeing_guidance_for_researchers_teams_and_supervisors_gold_silver_and_bronze_stand.

### Other Places to Access Resource

RESWELL tool kit: https://www.ucl.ac.uk/global-health/sites/global_health/files/res-well_toolkit.pdf.

Sexual Violence Research Initiative: https://www.svri.org/research-methods/researcher-trauma-and-safety.

Trauma Resource Initiative (they also have a free App called iChill): https://www.traumaresourceinstitute.com/.

### Emotionally Challenging Research Networks You Could Join

Researcher Wellbeing Group for graduate students and Early Career Researchers (international, based in UK): https://forms.office.com/Pages/ResponsePage.aspx?id=Ij1-N6FOLUKwrY_MiUBrnt81tb08TOtCupphEawJv7hUNk5CTjBYNzBGTjg4UllPQThWWUNIN0RHTC4u..

Research Resilience Community of Practice, primarily for graduate students and Early Career Researchers (international with leadership in Canada, UK, and Australia): https://www.researcherresilience.com/.

Emotionally Demanding Research Network Scotland: https://emotionalresearch.wordpress.com/.

Australian Advocacy for Safe and Ethical Research in Sensitive ConTexts Network: https://www.linkedin.com/company/aasert-network.

Challenging Research Network (international, based in UK): https://challengingresearch.org/.

### Want to Creating Change for Yourself and Other Researchers?

Join the Researcher Wellbeing Strategic Change Group: https://www.researchgate.net/publication/384838358_Researcher_Wellbeing_Strategic_Change_Group_RWSCG_About_Us. They have produced the following guidance:

- Wanting to instigate, influence or lead change around researcher wellbeing in your team, institution and/or discipline? Here is some guidance for Master, Doctorial students, and other Early Career Researchers: https://www.researchgate.net/publication/384838626_Wanting_to_instigateinfluencelead_change_around_researcher_wellbeing_in_your_team_insti.
- Wanting to instigate, influence or lead change around researcher wellbeing in your team, institution and/or discipline? Here is some guidance for principal investigators, supervisors, managers: https://www.researchgate.net/publication/384838171_Wanting_to_instigate_influence_or_lead_change_around_researcher_wellbeing_in_your_team.

## Footnotes

1 Skinner, Brance, et al. (2023) and Skinner, Bloomfield-Utting, et al. (2023) drew on Williamson et al.’s (2020) use of the term ‘clinical supervision’ to describe regular 1:1 meetings with a fully independent senior counsellor or psychotherapist to prevent vicarious trauma build up through guided confidential discussions. However, we acknowledge that the use of the term ‘clinical’ in the context of supervising researchers undertaking emotionally challenging research is problematic. This is because: (i) the level of training linked to the term ‘clinical’ varies in quality and length. In the UK, to be a ‘clinical psychologist’ requires formal postgraduate training (on a recognised 3–4 year DClinPsych course). Such practitioners tend to work with the most serious cases and can be costly. In contrast, most professionals who are ‘counsellors’ or ‘psychotherapists’, are not qualified as ‘clinical psychologists’ but have undertaken some form of training and accreditation, which can vary in length and quality. They can also take additional training to undertake ‘clinical supervision’, but this also varies in length and quality. (ii) The role of a ‘clinical supervisor’ in these professions is not the same as that being indicated as needed for researchers by the above authors. In the context of psychotherapy, for example, the focus of the ‘clinical supervisor’ is primarily the practice learning of the supervisee (see for example Milne & Dunkerley, 2010) not the wellbeing of the supervisee. (iii) The term ‘clinical’ could indicate to those in the medical profession that the level of need of the research is at a ‘clinically’ diagnosable level (e.g., ‘clinical’ depression, is an outdated way of inferring the condition is of a severity to warrant formal diagnosis and management by the health service). Instead of ‘clinical supervision’, Dickson-Swift et al. (2008) use the terms ‘professional supervision’ and ‘independent supervision’. They do not clearly define what is meant by these terms, other than someone outside of the university that can be used for debriefing and helping them to deal with their stress and distress. They do, however, support their argument for this service by referring to the requirement for supervision in codes of ethics linked to social work, counselling and psychotherapy (e.g., they referred to the 1992 American Psychological Association code of ethics, now APA, 2017). But the APA code talks primarily of supervision of more junior staff, for example, the APA (2017:8) states “Psychologists who delegate work to employees, supervisees, or research or teaching assistants […] take reasonable steps to […] authorize only those responsibilities that such persons can be expected to perform competently on the basis of their education, training, or experience, either independently or with the level of supervision being provided”. We have found a more detailed and directly relevant definition - of what Dickson-Swift et al. (2008), Skinner et al. (2023) and Williamson et al. (2020) appear to be wanting - given in the recommendations of the British Association for Counselling and Psychotherapy (BACP, 2018). BACP (2018) recommend supervision for “anyone working in roles that require regularly giving or receiving emotionally challenging communications” (p.24); to “sustain good practice throughout their working life”, to provide “regular and ongoing opportunities to reflect in depth about all aspects of their practice in order to work as effectively, safely and ethically as possible” *and* “sustains the personal resourcefulness required to undertake the work” (ibid.:22), they also state that it is preferable that it is done independently of line management by a counsellor with the training, expertise and experience to do so, confidentially “without blame or unjustified criticism” (p.24). Drawing on all the above, we suggest that *independent wellbeing supervision*, with an experienced and suitable counsellor or psychotherapist or psychologist (being mindful of the different qualifications and cost implications), that is independent of both the employer and the research project, and is regular, 1:1, confidential and focused on researcher wellbeing, is a clear way to label and define this service in relation to researchers undertaking emotionally challenging research.
